# The Secretome of the Inductive Tooth Germ Exhibits Signals Required for Tooth Development

**DOI:** 10.3390/bioengineering12020096

**Published:** 2025-01-21

**Authors:** Anahid A Birjandi, Paul Sharpe

**Affiliations:** Centre for Craniofacial and Regenerative Biology, Faculty of Dentistry, Oral & Craniofacial Sciences, King’s College London, London SE1 9RT, UK

**Keywords:** secretome, small extracellular vesicles, tooth regeneration, biotooth, cell-free tissue regeneration

## Abstract

Teeth develop from reciprocal signaling between inductive and receptive cells. The inductive signals for tooth development are initially in the epithelium of the developing branchial arch, but later shift to the underlying mesenchyme of a developing tooth germ. The inductive signals that are needed for tooth development have not yet been fully identified. Our lab previously provided a basis for bioengineering new teeth by separating the tooth germ’s epithelial and mesenchyme cells into a single cell population and recombing them. This approach, however, is not clinically applicable as the cells lose their inductive ability when expanded in vitro. In this study, we investigate whether the secretome and small extracellular vehicles (sEV) derived from inductive tooth germ mesenchyme can contribute to inductive signals required for tooth development. To address this, small extracellular vesicles and secretome were purified from inductive tooth germ mesenchyme and characterized. We investigated the proteome of sEV and proteome of inductive tooth germ mesenchyme and the impact of the culture condition and duration on the proteome. Additionally, we investigated the transcriptomic changes in tooth germ epithelium after treatment with sEV from inductive tooth germ mesenchyme. We show that culture duration of inductive tooth germ mesenchyme has an impact on the proteome of sEV purified from these cells. Similarly, culturing these cells in 2D and 3D environments results in different protein content. Proteome unique to sEV derived from inductive shows an association with multiple signaling pathways related to tooth development. Our RNASeq results show that treatment of tooth germ epithelial cells with small extracellular vesicles derived from inductive tooth germ mesenchyme results in an increased expression of some of the known odontogenic genes. Whilst further analysis is required to harvest the full potential of these sEV, our results suggests that extracellular vehicles contribute to signals required during tooth development, potentially through modulation of cellular metabolism and ECM organization.

## 1. Introduction

Tooth development requires a network of orchestrated signaling pathways that drives the reciprocal interaction between the dental epithelium and underlying neural crest-derived mesenchyme [1,2,3,4]. During the initial stages of tooth development, expression of *Msx1* and *Msx2* is regulated by the epithelial expression of *BMP4. Msx1* and *Msx2* are then expressed in the mesenchyme underlying the dental epithelium before and during tooth development [4,5,6]. This is followed by the shift of *BMP4* expression to the mesenchyme and the formation of a cell condensate [7,8,9]. The mesenchymal expression of *BMP4* subsequently modulates the expression of Wnt/B catenin signaling, which is crucial for further tooth development [10,11]. It is well established that the epithelium of developing tooth germ provides inductive signals until E11.5 of murine development. From this stage, the inductive capacity shifts to the mesenchyme. Our group had previously shown that early dental epithelium is able to induce tooth formation in a non-dental mesenchyme that has stem-cell-like properties. Similarly, tooth germ mesenchyme can induce tooth formation in a non-dental undifferentiated epithelium. This has been shown by recombining bone marrow stroma with inductive tooth epithelium or recombining adult gingival epithelial cells with embryonic tooth-inducing mesenchyme [12,13]. This approach allows teeth to be bioengineered from non-embryonic tooth cells using inductive signals. However, little is known about teeth inductive signals. BMP4 has been reported as a likely candidate for an inductive signal as its transition of expression from epithelium to mesenchyme coincides with the inductive signals shifting from epithelium to mesenchyme [8]. Parallel to the shift of *BMP4* expression, mesenchymal cells form a condensate which induces *BMP4* and expression of transcription factors *Msx1,2* and *Pax9* [14,15].

The successful bioengineering of teeth requires a better understanding of inductive odontogenic signals, particularly as the expansion of inductive cells in culture results in a loss of these signals [16]. In this study, we asked if paracrine signaling plays a role in tooth inductive signals. It is known that cell–cell communication and paracrine signaling play critical roles during organ development [17,18,19]. This paracrine signaling can be modulated by hormones or extracellular vesicles [20]. Extracellular vesicles are nano-sized membrane-bound particles that are secreted by cells into the extracellular space and play a vital role in communication between cells and their microenvironment. This is achieved by delivering bioactive molecules to target cells and, subsequently, influencing various biological processes [21,22]. There has been a great focus on cell secretome and secreted extracellular vesicles (sEV) due to their regenerative and therapeutic potential such as angiogenesis, lineage-specific differentiation, regulation of immune responses, and extracellular matrix organization [23,24,25]. In this study, we purify and characterize extracellular vesicles from inductive tooth germ mesenchyme and analyze its proteome. We analyzed differentially expressed genes in epithelial cells treated with sEV purified from inductive tooth germ to investigate whether these vesicles contribute to inductive signals required for tooth development.

## 2. Results

### 2.1. Purification of Extracellular Vesicles from Embryonic Tooth Germ

**Figure 1:**
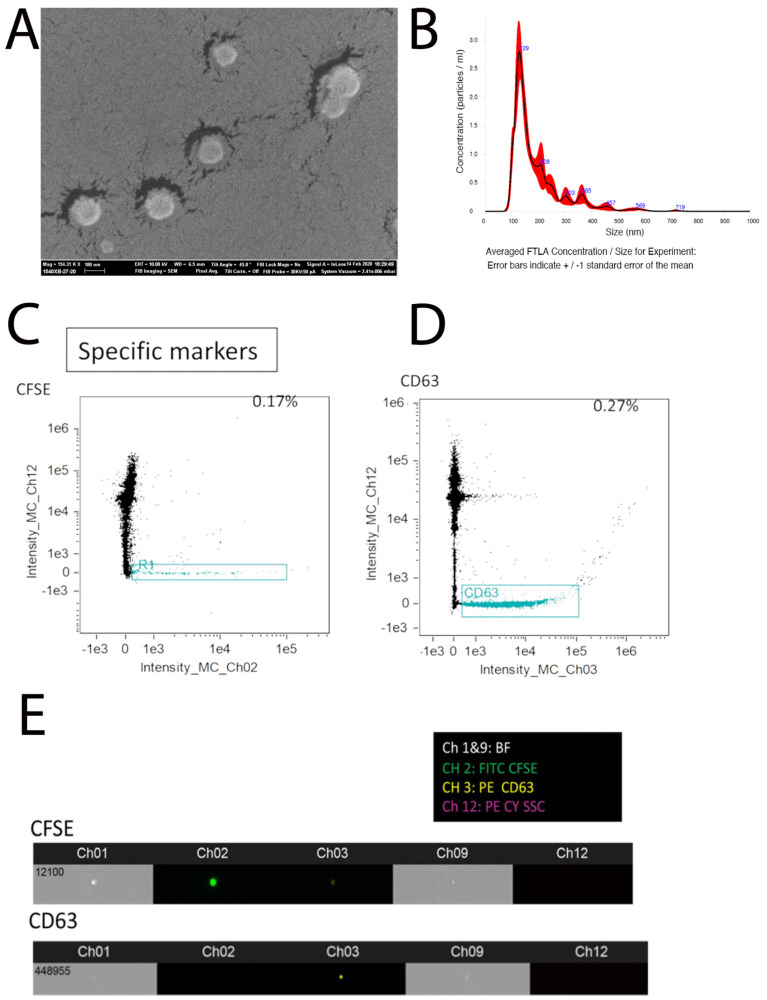
Characterization of s-EVs derived from murine embryonic tooth germ. Purified sEV were characterized using SEM (**A**). Concentration of the particles was measured with NTA (**B**). Advanced flow cytometry with Amnis image stream demonstrated a positive signal for CFSE (**C**) and CD63 (**D**) and their corresponding bead panel (**E**).

Small extracellular vesicles (sEV) were purified from tooth germ mesenchyme at E14.5 and the mesenchyme of first branchial arch at E10.5 (non-inductive mesenchyme control). Scanning electron microscopy demonstrated that isolated particles are around 100 nm large (Figure 1A). Nanoparticle tracking analysis showed the size of isolated particles to have a mean of 190.3 ± 4.0 nm and SD: 96.2 ± 1.1 nm and a concentration of 2.39 × 10^9^ ± 9.67 × 10^7^ particles/mL (Figure 1B). We used an advanced imaging cytometer to stain the isolated particles with a pan-exosome marker (CFSE) (Figure 1C) and an sEV-specific marker, tetraspanin CD63 (Figure 1D). These data confirmed that the isolated particles contained small extracellular vesicles. We refer to E14.5 tooth germ mesenchyme cells as inductive cells. Small extracellular vesicles (sEV) isolated from inductive mesenchyme of E14.5 tooth germ are referred to as i-sEV. sEV purified from non-inductive tissue will be referred to as sEV.

### 2.2. LC-MS/MS Analysis of Inductive Tooth Germ Mesenchyme Cells and Their Secretome

It is known that the epithelium of tooth germ provides inductive signals until E11.5, after which the inductive capacity shifts to the mesenchyme [12]. We used label-free proteomics to investigate the protein content of inductive cells (E14.5 mesenchymal cells) and their secreted extracellular vesicles. Since steps involved in the purification of sEV can result in the exclusion of some secreted proteins that could potentially have a role in tooth development, we also looked at the proteome of secretome purified from inductive mesenchymal cells. The proteomic groups were as follows:

1. E14.5 mesenchymal cells isolated from tooth germ;
2. Secretome purified from E14.5 mesenchymal cells;
3. s-EV purified from E14.5 mesenchymal cells.

**Figure 2:**
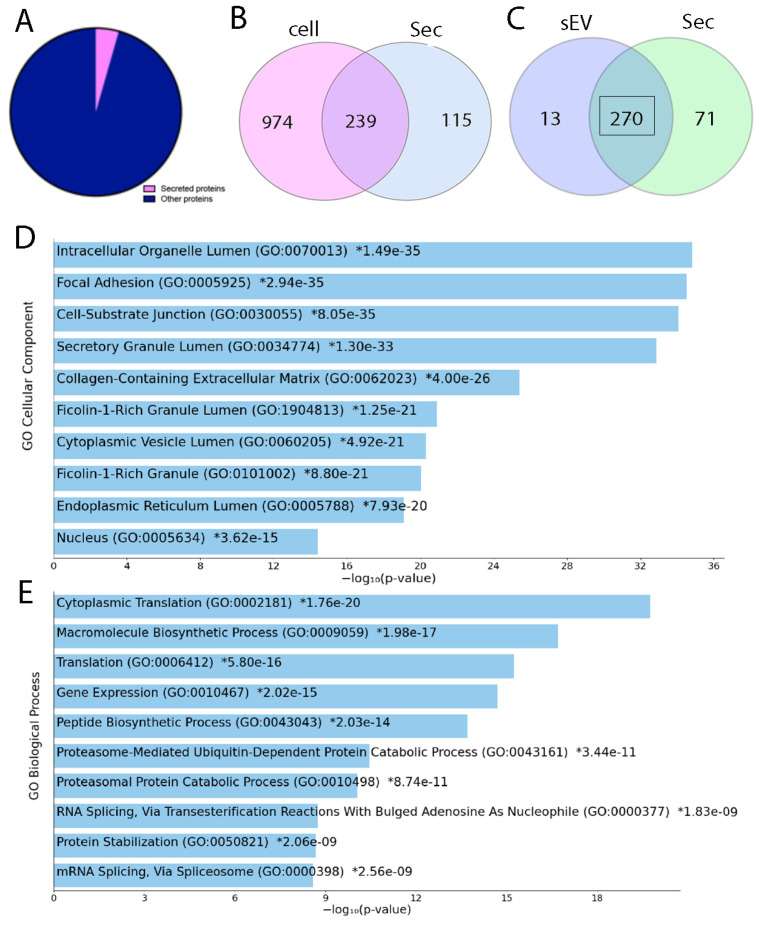
Shared and unique proteins in inductive mesenchyme cells and their secretome. Pie chart showing the ratio of secreted to non-secreted proteins in inductive cell proteome (**A**). Venn diagram showing the number of shared and unique proteins in inductive cells and their secretome (**B**) and unique proteins between the sEV and the whole secretome (**C**). Enrichr gene ontology of cellular component (**D**) and biological processes (**E**) associated with the shared proteins between sEV and secretome of inductive tooth germ mesenchyme.

After removing the contaminates across all the groups, MS/MS analysis identified 1186 proteins in inductive cells (Figure 2A) and 403 proteins across secretome groups. The distribution of shared and unique proteins between the cell and secretome can be seen in the Venn diagram (Figure 2B). Comparing the proteome of secretome and extracellular vesicles, we found that many of the identified proteins in the sEV are shared with the top 100 proteins in EV data bases (Figure 2C) (Supplementary Figure S1). Most of the proteins identified in sEV are shared with the secretome and associated with focal adhesion and secretory granule (Figure 2D). Enrichr Gene ontology demonstrated that these proteins are also associated with many biological functions such as cytoplasmic translation and peptide synthesis (Figure 2E). We found 13 proteins unique to sEV: B7-1a, Ccna2, Epg5, F11, Fbn1, fokIR, Lrp1, Nrip3, Rpl23, SCO5089, Tdg, TPM2, and TrpG.

### 2.3. Impact of Culture Conditions on the Proteome of sEV Purified from Inductive Mesenchyme

It is known that the cell culture environment (2D vs. 3D) can have an impact on cell signaling [26]. Therefore, we next investigated the impact of a 2D and 3D culture environment on the proteome of sEV derived from inductive tooth germ mesenchyme (i-sEV). The study groups were as follows:

1. i-sEV derived from E14.5 tooth mesenchyme cultured for 24 h in 3D culture wells;
2. i-sEV derived from E14.5 tooth mesenchyme cultured for 24 h in 2D culture wells.

**Figure 3:**
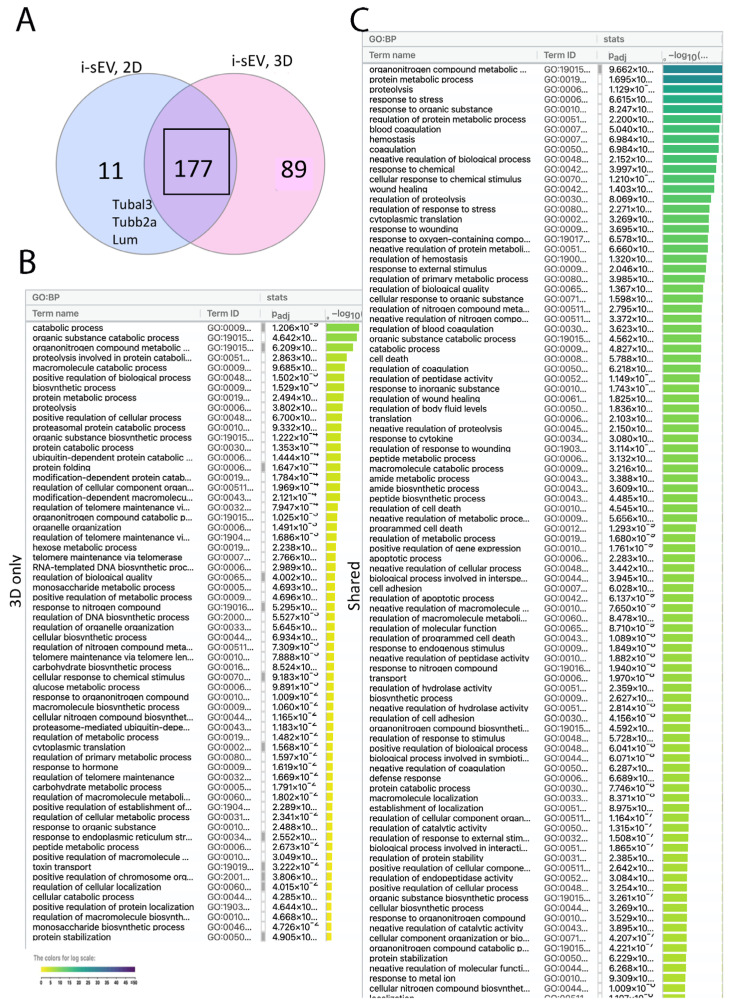
Impact of culture condition on the proteome on sEV derived from inductive tooth germ mesenchyme. Venn diagram showing the number of shared and unique proteins between sEV derived from inductive tooth mesenchyme cultured for 24 hr in 2D and 3D environments (**A**). GO enrichment analysis by g: profiler showing biological function unique to 3D i-sEV (**B**) and g: profiler enrichment analysis showing biological function of shared proteins between 2D and 3D i-sEV (**C**).

We identified 11 proteins unique to i-sEV cultured in a 2D environment such as members of Ribosomal subunit, heterogenous nuclear proteins, Tubulin A and B, and lumnican, a member of SLRP that binds collagen fibrils (Figure 3A). The 89 proteins unique to i-sEV cultured in 3D environment were enriched in catabolic process and glucose metabolic process (Figure 3B). There were 177 proteins shared between 2D and 3D i-sEV, with enrichment in protein metabolic process, proteolysis, and hemostasias (Figure 3C).

### 2.4. Proteome Unique to Inductive sEV Shows Association with Multiple Signaling Pathway Related to Tooth Development

It is known that tooth inductive signals are lost upon in vitro expansion of these cells [16]. Therefore, we comparted the proteome of i-sEV purified from E 14.5 mesenchyme after different culture durations to identify proteins unique to i-sEV. We used sEV derived from mesenchyme of E10.5 branchial arch as a non-inductive control. The study groups for this experiment were as follows:

1. sEV derived from E14.5 tooth mesenchyme cultured for 24 h in 3D culture wells (i-sEV 24 h);
2. sEV derived from E14.5 tooth mesenchyme cultured for 5 days in 3D culture wells (i-sEV 5 days or cultured i-sEV);
3. sEV derived from mesenchyme of E10.5 branchial arch cultured for 24 h as non-inductive control (sEV).

**Figure 4:**
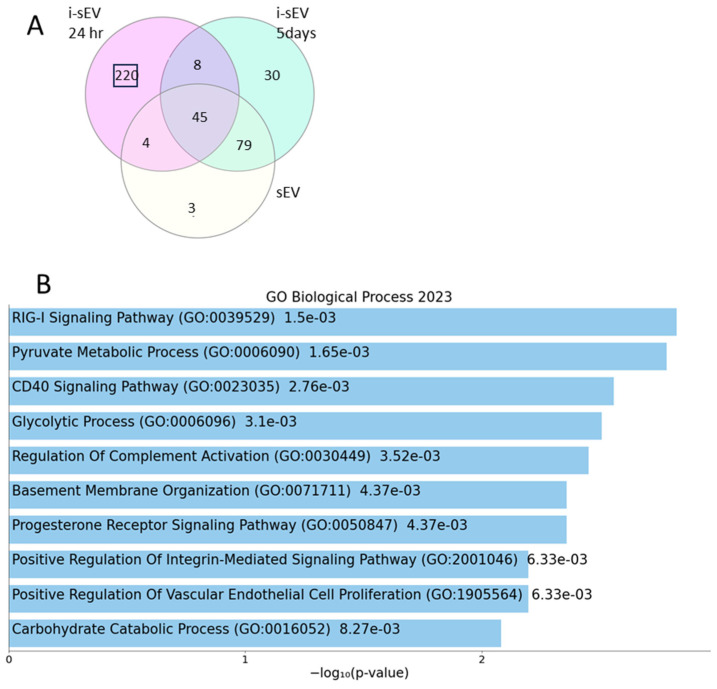

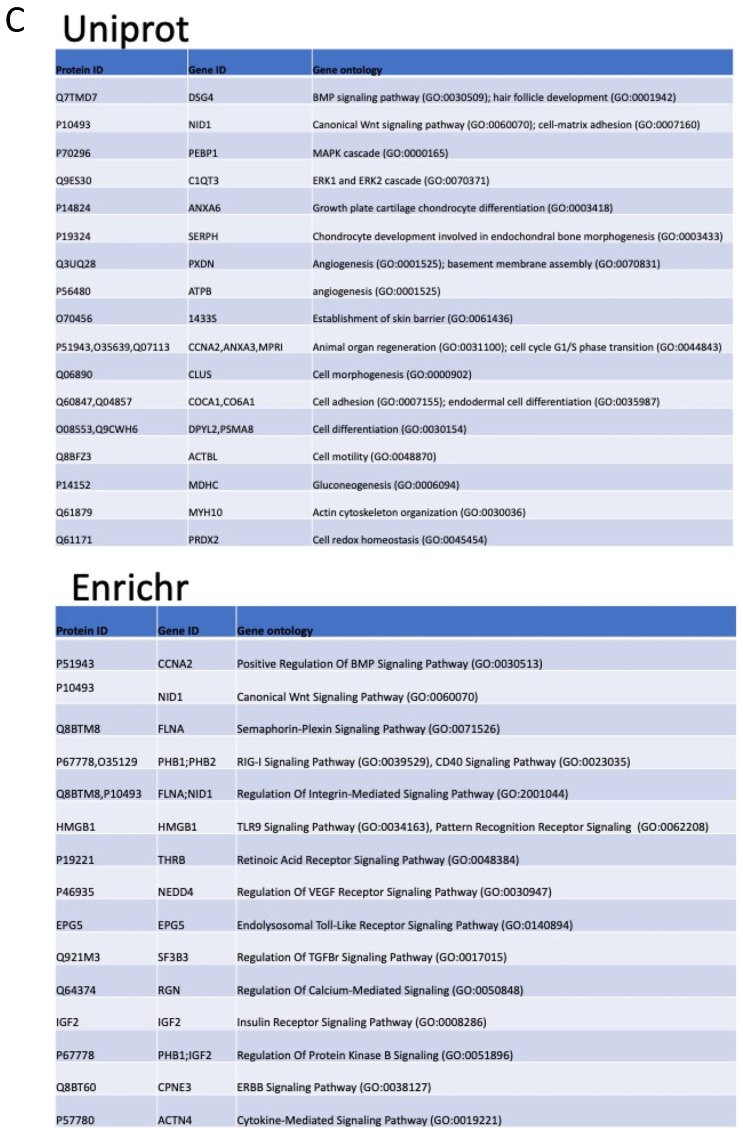
Proteome of inductive tooth mesenchyme sEV. Venn diagram showing number of shared and unique proteins between sEV derived from inductive tooth mesenchyme cultured for 24 h or 5 days, and non-inductive mesenchyme from E10.5 branchial arch (**A**). Enrichr Gene ontology of selected biological function associated with 220 proteins unique to sEV derived from short-cultured inductive tooth mesenchyme (i-sEV) (**B**). Table demonstrating identified proteins and their associated signaling pathway from Uniprot and Enrichr (**C**).

We identified 220 proteins unique to 24 h cultured i-sEV (Figure 4A). Gene ontology of these proteins demonstrates association with RIG signaling pathway, CD40 signaling pathway, basement membrane organization, and angiogenesis as well as signaling pathways known to be involved in tooth development such as BMP (Figure 4B). Using Uniprot and Enrichr, we identified proteins associated with these signaling pathways. These results demonstrate that the proteome unique to i-sEV cultured for only 24 hr contains proteins that are associated with signaling pathways known to be important in tooth development such as DSG4, NID1, and FLNA (Figure 4C).

### 2.5. Treatment of Tooth Germ Epithelium with sEV from Inductive Mesenchyme

We found that the proteome of 24 h cultured i-sEV contains proteins that are important in tooth development. Therefore, we asked whether the treatment of tooth germ epithelium with these i-sEV results in any transcriptomic changes. Using Bulk RNA sequencings, we investigated differentially expressed genes in the following groups:

1. E14.5 tooth germ epithelium treated with i-sEV (derived from 24 h cultured E14.5 tooth germ mesenchyme),
2. E14.5 tooth germ epithelium treated with cultured i-sEV (derived from 5-day-cultured E14.5 tooth germ mesenchyme),
3. untreated E14.5 tooth germ epithelium as our negative control,
4. inductive epithelium from E10.5 branchial arch as our positive control.

**Figure 5:**
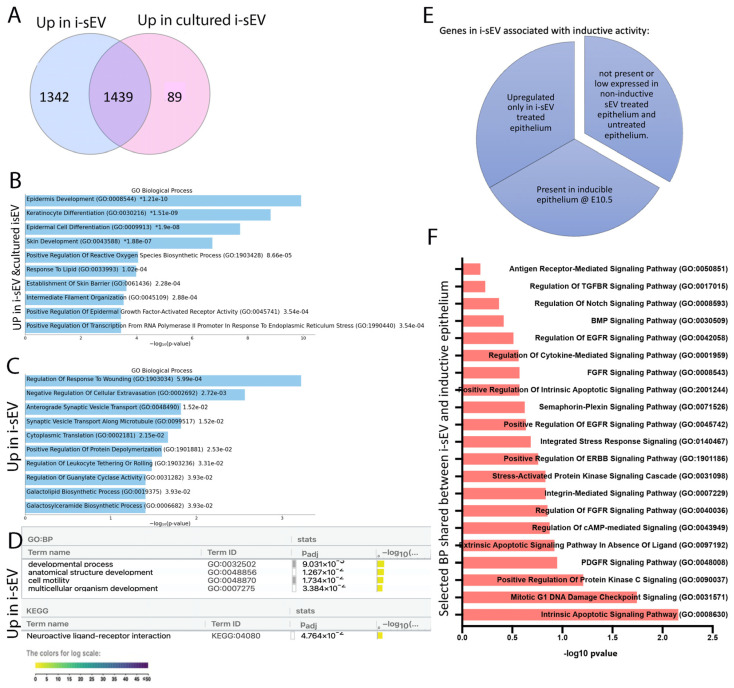
Treatment of tooth germ epithelium with sEV from inductive mesenchyme. Venn diagram shows shared and unique upregulated genes between epithelium treated with i-sEV and epithelium treated with cultured i-sEV (**A**) and Enricher Gene ontology of their shared genes (**B**). GO Enrichr demonstrates biological functions upregulated only in i-sEV treatment (**C**). G profiler demonstrates enriched biological functions and KEGG pathway upregulated only in i-sEV treatment (**D**). Selected biological functions from Enricher Gene ontology of shared upregulated genes between inducible epithelium and i-sEV treatment (**E**,**F**).

The investigation of differentially expressed genes (DEG) in i-sEV-treated epithelium and untreated epithelium showed upregulation of skin development, epidermal cell differentiation, and keratinization (Supplementary Figures S2 and S3). Since the proteome of i-sEV was significantly different than 5-day-cultured i-sEV, we hypothesized that treatment with these two groups of sEV results in different transcriptomic changes in epithelium. To identify upregulated genes that are uniquely associated with i-sEV, we looked at shared and unique upregulated genes in treatment with i-sEV and 5-day-cultured i-sEV (Figure 5A). We found that upregulation of epidermis development and keratinocyte differentiation are shared between i-sEV and 5-day-cultured i-sEV and are, thus, not unique to i-sEV treatment (Figure 5B). By contrast, response to wound healing and regulation of leukocyte tethering as well as enrichment in cell motility and multicellular development are unique to i-sEV treatment (Figure 5D).

To identify genes in i-sEV that are associated with inductive activity, we looked at genes that are upregulated only in epithelium treated with i-sEV and present in inductive epithelium of E10.5 branchial arch. For this purpose, we looked at normalized gene counts in studied groups and selected the genes that had high counts in inductive epithelium and i-sEV-treated epithelium and were also not present or expressed at low counts in non-inductive sEV-treated epithelium and untreated epithelium (Figure 5E). Selected biological functions associated with these genes are PDGFR signaling, apoptotic signaling pathway, and the regulation of protein kinase C signaling (Figure 5F). The list of all genes can be seen in Supplementary Table S1.

### 2.6. Treatment of Tooth Germ Epithelium with Secretome from Inductive Mesenchyme

Steps involved in the purification of sEV can result in exclusion of some secreted proteins that could potentially have a role in tooth development. Therefore, we next investigated the effect of treatment of tooth germ epithelium with inductive secretome derived from E14.5 tooth germ mesenchyme. We call this group i-sec-treated epithelium.

**Figure 6:**
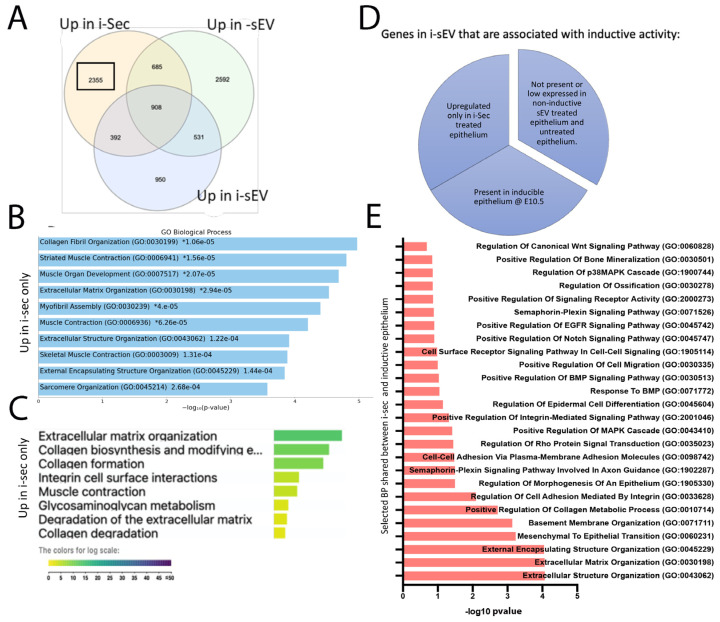
Treatment of tooth germ epithelium with secretome from inductive mesenchyme. Venn diagram shows shared and unique upregulated genes between inductive secretome treatment, inductive sEV treatment, and long-culture sEV treatment (**A**). GO Enrichr demonstrates biological functions upregulated only in i-Sec treatment (**B**). G profiler demonstrates enriched pathways upregulated only in i-sec treatment (**C**). Selected biological functions from Enricher Gene ontology of shared upregulated genes between inducible epithelium and i-sec-treated epithelium (**D**,**E**).

Differentially expressed genes after treatment with i-sec showed upregulation of various biological functions such as wound healing, skin development, leukocyte migration, and Integrin binding (Supplementary Figure S4). We looked at shared and unique upregulated genes between i-sEV and i-Sec to identify factors uniquely upregulated in i-Sec and to see if any of these are required during tooth development (Figure 6A). Collagen fibril organization and enrichment in ECM organization, leukocyte migration and activation, chemotaxis, and the regulation of EMT are associated with genes upregulated only in i-Sec (Figure 6B,C).

To identify potential inducible factors in secretome that may be lost during sEV purification, we looked at genes that are upregulated in epithelium treated with i-sec and present in inductive epithelium of E10.5 branchial arch. For this purpose, we looked at normalized gene counts in all studied groups and selected those that were high in i-sec-treated epithelium and inductive epithelium but low in non-inductive sEV-treated and untreated epithelium (Figure 6D). Selected biological functions associated with these genes were ECM organization, basement membrane organization, cell adhesion by Integrins, and the regulation of epithelial morphogenesis (Figure 6E). The list of genes can be seen in Supplementary Table S2.

### 2.7. Generation of Bioengineered Teeth Using Secretome of Inductive Tooth Germ Mesenchyme

**Figure 7:**
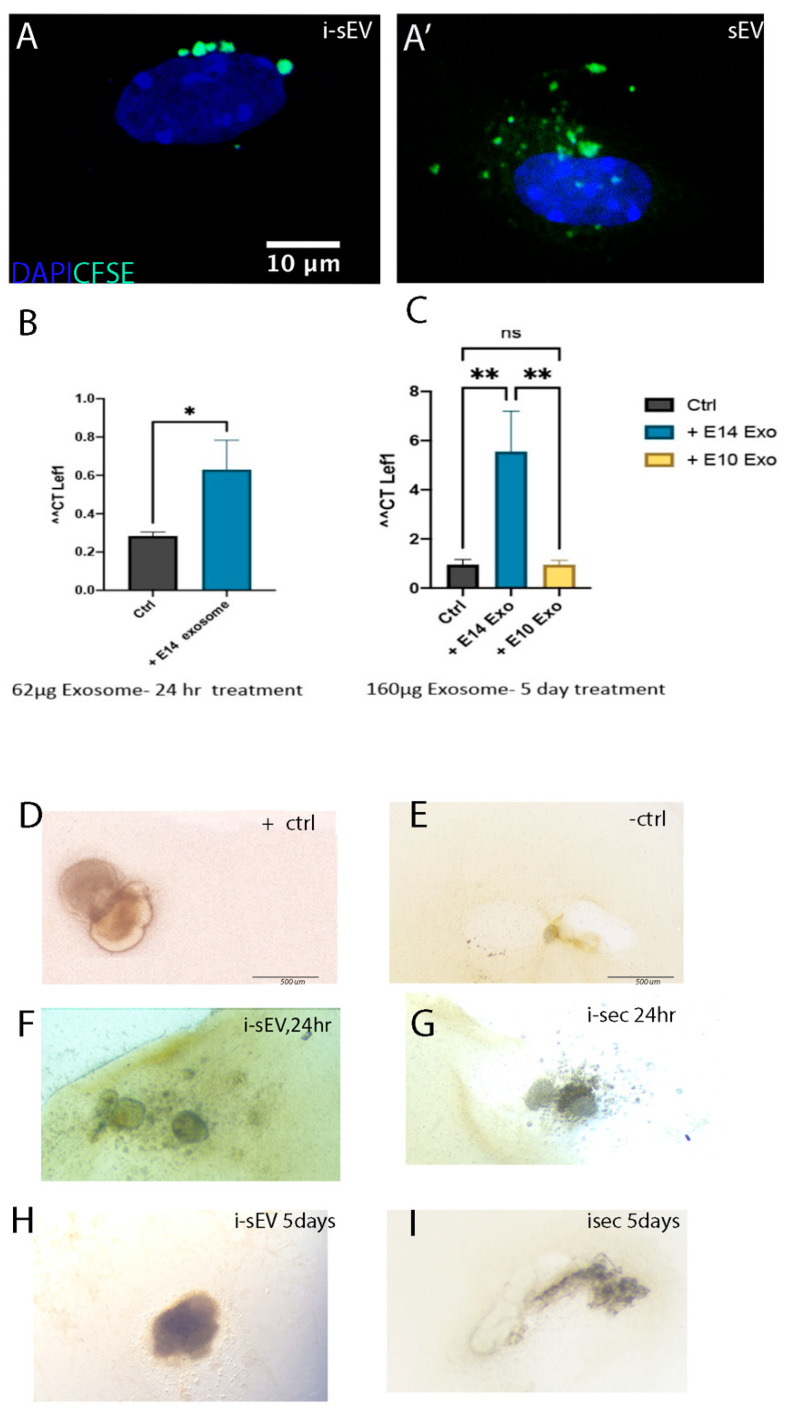
Generation of bioengineered teeth using i-sEV and i-Sec. CFSE-labelled i-sEV and s-EV are taken up by E10.5 branchial arch mesenchyme after 24 h. (**A**,**A′**) Expression of *Lef1* after treatment of tooth germ epithelium with i-sEV for 24 h (**B**) and 5 days (**C**). Recombination of fresh E14.5 mesenchyme cells with E13.5 tooth germ epithelial cells (**D**). Recombination of E10.5 branchial arch mesenchyme with E13.5 tooth germ epithelial cells (**E**). Recombination of i-sEV-treated epithelial cells for 24 h with E10.5 branchial arch mesenchyme (**F**). Recombination of i-sEV-treated epithelial cells for 5 days with E10.5 branchial arch mesenchyme (**G**). Recombination of i-Sec-treated epithelial cells for 24 h with E10.5 branchial arch mesenchyme (**H**). Recombination of i-Sec-treated epithelial cells for 5 days with E10.5 branchial arch mesenchyme (**I**). *p* < 0.002 (**), *p* < 0.033 (*), *p* > 0.12 (ns).

We noticed that Integrin signaling pathway was associated with i-sEV proteome and was also upregulated in i-sEV- and i-Sec-treated epithelium. Integrin signaling pathway and focal adhesions interact with the Wnt signaling pathway, which is crucial during tooth development [27,28]. Therefore, we treated tooth germ epithelium with i-sEV for 24 h and 5 days to see if this results in the promotion of Wnt signaling. Our data demonstrate upregulation of *Lef1* expression, particularly after the 5-day treatment of epithelium with i-sEV (Figure 7A–C). Having seen the increased Wnt signaling in i-sEV-treated epithelium and changes in transcriptome of tooth germ epithelium with i-sEV and i-Sec treatment, we asked if these changes were sufficient to induce tooth induction ex vivo.

As used in previous work from our lab, we recombined E14.5 mesenchyme cells with E13.5 tooth germ epithelial cells as the positive control (Figure 7D). For the negative control, we recombined E10.5 mesenchyme cells with E13.5 tooth germ epithelial cells (Figure 7E). Next, we recombined non-inductive tooth germ mesenchyme from E10.5 branchial arch with tooth germ epithelium treated with i-sEV for 24 h (Figure 7F) and for 5 days (Figure 7H). For secretome groups, we recombined non-inductive tooth germ mesenchyme with tooth germ epithelium treated with i-sec for 24 h (Figure 7G) and 5 days (Figure 7I) with non-inductive tooth germ mesenchyme from E10.5 branchial arch. We found that, although significant transcriptome changes resulted after treatment of tooth germ epithelium with i-sEV and i-Sec, these changes were not sufficient to induce complete tooth induction ex vivo.

## 3. Discussion

Gradients of signaling molecules and cell–cell and cell–matrix interactions modulate mesenchymal cell differentiation during organ formation such as hair, feather, and tooth [29,30,31,32,33]. Tooth bud mesenchyme is able to induce tooth formation in a non-dental undifferentiated epithelium. This has been shown by recombining bone marrow stroma with inductive tooth epithelium and by recombining adult gingival epithelial cells with embryonic tooth-inducing mesenchyme [12,13]. In this study, we investigated whether the secretome of inductive tooth germ mesenchyme can contribute to the inductive signals required during tooth development. Therefore, small extracellular vesicles (sEV) were isolated from mouse embryonic tooth germ (Figure 1). Our proteomic analysis demonstrates that many of the proteins identified in purified vesicles were amongst the top 100 proteins identified in the extracellular vesicle database such as GAPDH, FN1, and ANXA1, confirming that the isolated particles in our hand contained sEV (Supplementary Figure S1). We showed that the secretome from inductive tooth germ mesenchyme has many shared proteins with its parental cells. Similarly, most of the proteins identified in sEV are shared with the secretome, suggesting that many of the functions exerted by sEV are shared with the secretome (Figure 2). Unique proteins identified only in sEV are also present in the sEV database (Veisclepedia) such as Ccna1, F11, FBn1, Lrp1, RPL23, Tdg, and Tpm2 [34].

It is known that the cell culture environment can regulate cell function and signaling [26,35,36,37]. We found that sEV purified from the 3D culture of inductive tooth germ mesenchyme had a higher number of proteins than sEV purified from the 2D culture of these cells (Figure 3). Proteins unique to sEV purified from the 2D environment (Tubulin and Lumincan) were mostly associated with microtubule assembly. This can be attributed solely to culture condition. It also suggests that a 24 h culture duration can be sufficient to impact parental cells, resulting in a different proteome of purified sEV. The inductive ability of E14.5 tooth germ mesenchyme has been shown before [38]. Our group has previously shown that embryonic inductive tooth germ cells rapidly lose their inductive capacity following in vitro expansion [16]. Here, we found that the proteome of i-sEV isolated from expanded inductive cells is more similar to that of sEV purified from non-inductive mesenchyme (Figure 4). This suggests that the in vitro expansion of inductive cells can impact the cell secretome and, subsequently, their paracrine signaling. This is also reflected in the difference between the transcriptome of epithelium treated with short cultured and long-cultured i-sEV (Figure 5). Interestingly, Enrichr Gene ontology of proteome unique to i-sEV showed an association with Retinoic acid-inducible gene I signaling (RIG-I) pathway, CD40 signaling pathway, basement membrane organization, Integrin signaling, and angiogenesis. RIG-I is involved in pattern recognition signaling and, similar to Retinoic acid, is required during embryonic and tooth development [39,40,41,42]. Similarly, the interaction between Fibronectin and Integrins is essential for tooth development [43]. Fibronectin and β1 Integrin are highly expressed in dental mesenchyme. The abundant expression of β1 Integrin has been detected in regions of the basement membrane and in mesenchymal cells [44,45]. Glycolytic process and other signaling pathways known to be involved in tooth development such as BMP were also detected in i-sEV proteome [46,47,48].

Additionally, the transcriptome of i-sEV-treated tooth germ epithelium exhibited response to wounding, glycolipid biosynthesis, and enrichment in anatomical structure development. This suggests that the content of i-sEV can exert unique changes in tooth germ epithelium (Figure 5C,D). We noticed that some of the genes that were only upregulated in i-sEV-treated epithelium were also present in the inductive epithelium of E10.5 branchial arch. These shared genes were associated with protein kinase C signaling, PDGFR, FGFR signaling, and BMP signaling, all of which have known important roles during tooth development [49,50,51]. It is, therefore, plausible to suggest that genes/signaling molecules that are upregulated only in i-sEV-treated epithelium and are present in the inductive epithelium can serve as candidate factors that contribute to tooth inductive signals (Supplementary Table S1).

In our study, signaling pathways such as Wnt, BMP, and Integrin signaling were associated with i-sEV proteome and transcriptome of i-sEV-treated epithelium. It is known that Integrin-linked kinases can regulate the Wnt/B-catenin pathway. Some of this interaction is through the modulation of Wnt signaling in response to ECM stiffness [27,28,52,53]. This suggests that Integrin signaling in the secretome can contribute to mesenchymal condensation and the subsequent expression of Wnt signaling. We show that the 5-day treatment of epithelium with i-sEV results in the upregulation of the Wnt signaling pathway, a pathway crucial for tooth development [54]. However, the promotion of the Wnt signaling pathway and the presence of the candidate inducible factors in inductive sEV and secretome (i-sEV and i-Sec) may not be sufficient to induce tooth development ex vivo. This was seen when the recombination of inductive mesenchyme cells with embryonic epithelial cells resulted in tooth formation ex vivo in the control group, but the recombination of i-sEV-treated or i-sec-treated epithelium with non-inductive mesenchyme failed to do so (Figure 7). There are couple of explanations for this. One could be that the epithelium in our study may have lost the ability to receive the inductive signals and was subsequently unable to participate in tooth induction. Another hypothesis is that tooth development requires a well-orchestrated signaling pathway [55,56,57]. Our secretome was isolated from inductive tooth germ at E14.5. At this stage, dental mesenchyme cells segregate into dental papilla and dental follicle lineages. Many of these changes rely on direct cellular interaction, and paracrine signaling may only play a small part in this process. It has been shown that a group of migratory MSX1+ Sox9+ from the dental niches directly contribute to dental papilla [15,58]. In our study, MSX1 was upregulated in both i-sEV- and i-Sec-treated epitheliums. Similarly, proteins associated with Wnt and BMP signaling were identified in proteome unique to i-sEV and i-sec, all of which are necessary but not sufficient for the induction of tooth formation ex vivo. Additionally, many proteins and secreted factors can be excluded during the steps of sEV purification [59,60,61].

Biological functions associated uniquely with epithelium treated with inductive secretome suggest that the secretome of inductive tooth germ mesenchyme has unique factors, different to i-sEV, that can contribute to tooth development (Figure 6). Treatment of epithelium with inductive secretome results in more significant changes in transcriptome. This can have an unintended impact on the treated epithelium. Therefore, the additional modification of secretome may be required to control the level of signaling required for tooth development. This suggests that using sEV rather than the whole secretome can be a more targeted and strategic approach in the investigation of inductive tooth signals. It is possible that increasing the concentration of sEV used for epithelial treatment or the duration of treatment could enhance the odontogenic signals; however, maintaining the viability of epithelial cells during longer treatment may be challenging. Furthermore, sEV heterogeneity needs to be considered, as it will be difficult to eliminate the batch effect during different sEV treatments when a higher concentration of sEV is required. Additionally, co-purification of plasma proteins and lipids is well documented during sEV isolation. Many types of secreted molecules can exert biological effect and should be considered [42,62,63,64].

Our study demonstrates that the secretome from inductive tooth germ mesenchyme contributes to signals required during tooth development, potentially through the modulation of cellular metabolism and ECM organization. However, these signals are not sufficient to induce tooth formation ex vivo. This suggests that treatment of epithelium with the secretome of inductive mesenchyme has the potential to restore some of the inductive signals. However, at least on the scale of this murine study, it does not fully replace the signals from inductive mesenchyme that are essential for tooth development. This study provides important insight into the potential of tooth germ secretome. Further investigations are required to fully harvest this potential for whole-tooth bioengineering.

## 4. Materials and Methods

### 4.1. Cell Culture

Small extracellular vesicles (sEV) were purified from murine embryonic tooth germ and branchial arch. These were harvested from wildtype mouse in a CD-1 background and bred in-house in the animal facility of King’s College London. Animals were sacrificed by Schedule 1 by cervical dislocation. CD1 embryonic mesenchyme of first branchial arch at E 10.5 (non-inductive mesenchyme control) and the mesenchyme of tooth germ at E14.5 were trimmed from any surrounding tissue. The tooth germs were incubated in DPBS (−) containing 1.2 U/mL Dispase II (Roche, Basel, Switzerland) for 15 min at RT. Epithelium and mesenchyme were separated with fine needles. Following separation of epithelium and mesenchyme, the mesenchyme of these tissues was digested to prepare single-cell suspension. All procedures were in accordance with the UK Animals (Scientific Procedures) Act (1986). All animal experiments were approved by the Institutional Animal Care and Use Committees King’s College London (PPLP5F0A1579). This study conforms with ARRIVE guidelines.

### 4.2. Purification and Characterization of Small Extracellular Vesicles

Throughout this study, small extracellular vesicles (sEV) were purified from single-cell suspensions of tooth germ mesenchyme. We used complete media DMEM with 10% exosome depleted FBS (Thermofisher, Waltham, MA, USA, A2720803). The study groups were as follows:

1. sEV derived from E14.5 tooth germ mesenchyme cultured for 24 h in 3D culture wells;
2. sEV derived from E14.5 tooth germ mesenchyme cultured for 24 h in 2D culture wells;
3. sEV derived from E14.5 tooth germ mesenchyme cultured for 5 days in 2D culture wells.

For every experiment, 3 batches of small extracellular vesicles (sEV) were purified. EV purification was performed by differential centrifugation followed by ultracentrifugation (Beckman Coulter, Brea, CA, USA) using TLA100.3 rotor at 4 °C. The resultant pellet was resuspended in exosome-depleted PBS and used for further characterization or kept in *−*80 until further application. For 3D culture, a 96-well culture plate with “u” bottom was used. For the secretome group, secretome was harvested from single-cell suspension of E14.5 tooth germ mesenchyme cells cultured for 24 h in 3D environment and subject to gradient centrifuge ultracentrifuge. Purified secretome was kept at *−*80 until further application.

For scanning electron microscopy, sEV were fixed in 4% paraformaldehyde (PFA). Particles were spotted on a clean flat silicon surface from isopropanol suspension in a 10 µL volume, dried, and imaged by in-lens detector in a Karl Zeiss XB1540 SEM with voltage 10 keV (Thermo Scientific Inc.). To determine size and concentration of purified sEV, nanoparticle tracking analysis (NTA) was performed with NanoSight LM10 (Malvern Instruments, Worcestershire, UK) according to manufacturer’s instruction and NanoSight NTA 3.2 software [65]. Advanced nano flow cytometry (FCM) was used to detect sEV-specific markers. Briefly, purified sEV were incubated with CFSE and CD63 (mAbs; BioLegend, San Diego, CA, USA) for 30 min and subject to Amnis image stream. Buffer only and dye only (of each channel) were used as control.

For images of sEV uptake, airyscan in confocal microscopy with LSM980 Zeiss, Oberkochen, Germany was used.

For all experiments (proteomics and RNA seq), a volume corresponding to an equal amount of protein (100 μg) was used per sample [66]. 

### 4.3. Quantitative PCR

For *Lef1* expression, epithelial cells from E14.5 tooth germ were dissociated, cultured, and plated in triplicates in 24-well plates and incubated using standard culture medium (37 °C, 5% CO2/95% air, 100% humidity). Following 24 h, cultured cells were treated with sEV from different groups at a volume corresponding to 60–150 ug protein. After 24 h, cells were lysed with Trizol for extraction of RNA. Real-time qPCR using Sybr Green (Roche, Basel, Switzerland) on a Rotor-Gene Q cycler (Qiagen, Hilden, Germany) system was performed as recommended by the manufacturer’s instructions [67]. Beta-actin was used as the reference gene (Forward-GGCTGTATTCCCCTCCATCG, Reverse-CCAGTTGGTAACAATGCCTGT) and Lef1 expression levels as the read-out for Wnt pathway activity (Forward-TGACTCTCCTTCCAGATCCCA, Reverse-TGCCCACACTAGGCTGACA). Reactions were performed in triplicate and relative changes to the reference gene were calculated by the 2^−∆∆CT^. Normal distribution was tested with Shapiro–Wilk test and statistical significance was tested with one-way ANOVA and Tukey multiple comparisons using GraphPad Prism 9. For comparison of differentiation marker expression, the Kruskal–Wallis test was used. Adjusted *p* values reported in graphs according to New England Journal of Medicine guidelines: *p* < 0.001 (***), *p* < 0.002 (**), *p* < 0.033 (*), *p* > 0.12 (ns).

### 4.4. Proteomics

Secreted extracellular vesicles and the secretome were purified as described. “Media only” samples were used as control group, subjected to same purification and ultracentrifugation steps, and resuspended in exosome-depleted PBS, similar to experimental groups. This was to eliminate the contaminants that were present in the media from the analysis. All samples were initially loaded into precast Tris-Bis gel for short resolution to ‘stack’ the protein complement and purify the contaminants prior to protein digestion. Samples were then resolved for 20 min and stained overnight in a colloidal protein stain. Enzymatic digestion and peptide extraction were performed according to protocol. Peptide sample was resuspended in resuspension buffer (Thermo Fisher Scientific, Waltham, MA, USA) and resolved by reversed phase chromatography. The eluate was ionized by electrospray ionization using an Orbitrap Fusion Lumos (Thermo Fisher Scientific, Waltham, MA, USA) operating under Xcalibur v4.3. Orbitrap spectra (FTMS1) were collected at a resolution of 120,000 over a scan range of *m*/*z* 375–1600 with an automatic gain control (AGC) setting of 4.0 × 10^5^ (100%) with a maximum injection time of 35 ms. Monoisotopic precursor ions were filtered using charge state (+2 to +7) with an intensity threshold set between 5.0 × 10^3^ and 1.0 × 10^20^ and a dynamic exclusion window of 35 s ± 10 ppm. MS2 precursor ions were isolated in the quadrupole set to a mass width filter of 1.6 *m*/*z*. Ion trap fragmentation spectra (ITMS2) were collected with an AGC target setting of 1.0 × 10^4^ (100%) with a maximum injection time of 35 ms with CID collision energy set at 35%. Database search was performed according to manufacturer’s instruction [66,67]. Proteome Discoverer was used for data base searching (ThermoScientific; v2.5) in a label-free quantitative workflow. Raw data were processed and searched using the Mascot search algorithm v2.6.0; (www.matrixscience.com accessed on 5 July 2024) and the Sequest search algorithm against the Uniprot mouse Taxonomy database [68]. For analysis, Scaffold software v5.1.2 (proteomesoftware.com, accessed on 5 July 2024) was used. After filtering entries shared with media control, experiment-wide grouping with protein cluster with a peptide threshold of 95% minimum was performed. For functional enrichment analysis, G profiler was used with significance threshold set at Bonferroni correction and Term size set at 50. For comparison of inductive vs. non-inductive proteome, we compared the protein identified in inductive sEV for both 2D and 3D from label-free run with those identified in long-culture i-sEV and non-inductive sEV (e10.5) using isobaric mass tagging (TMT; Thermo Fisher Scientific). This was achieved by considering presence or absence of any value in the TMT Reporter ion values and the TIC values from label-free run. There were some levels of sample loss due to normalization in TMT run. Particularly, observing the variation in different batches of sEV and across other groups, it would be difficult to account for 1:1 ratio without losing protein in some samples. Therefore, for the second run, where groups included sEV, secretome, and cell pellet, we aimed to perform label-free proteomics. To identify proteins unique to i-sEV, we comparted the proteome of sEV derived from short (24 h) culture of inductive cells (this includes 2D and 3D and so-called i-sEV) with that of sEV derived from long (5-day) culture of inductive cells. sEV derived from mesenchyme of E10.5 branchial arch as a non-inductive control [66,67,68].

### 4.5. RNA Sequencing

Tooth germ epithelium was dissected and treated with sEV derived from inductive and non-inductive tooth germ mesenchyme for 24 h. Each sEV treatment was a pool of 3 batches of purified sEV. RNA was isolated with Trizol (Thermo Fisher Scientific, Waltham, MA, USA) and Qiagen Mini kit (Hilden, Germany). After assessment of quality and quantity, we sent for Bulk Sequencing to Novogene UK Ltd. (Cambridge, UK) using Illumina NovaSeq 6000 instrument. Sample QC was carried out according to Novogene UK protocol [69]. In brief, mRNA was purified using poly-T oligo-attached magnetic beads. For the non-directional library, it was ready after end repair, A-tailing, adapter ligation, size selection, amplification, and purification. Analysis pipeline was carried out according to Novogene protocol. For the directional library, it was ready after end repair, A-tailing, adapter ligation, size selection, USER enzyme digestion, amplification, and purification. Libraries were used according to Novogene protocol. For quantification of gene expression level, the feature Counts v1.5.0-p3 was used to count the read numbers mapped to each gene, and FPKM of each gene was calculated [69,70]. For differential expression analysis, EdgeR was used. The *p* values were adjusted using the Benjamini and Hochberg method. Corrected *p*-value of 0.05 and absolute fold change of 2 were set as threshold for significantly differential expression. For gene ontologies, Enrichr biological function and bioplanet pathways were used. For biological function, we used −log10 *p*-value for the selected GO terms and graphed those with −log10 *p* value 1.5. For functional enrichment analysis, G profiler was used with significance threshold set at Bonferroni correction and Term size set at 50. To identify potential inducible candidates, we looked at heatmap counts of genes that are higher in inducible epithelium in comparison with untreated epithelium (or have no count in untreated epithelium). These genes should be high in i-sEV-treated epithelium but demonstrate lower counts in non-inducible sEV-treated epithelium [69,70].

### 4.6. Generation of Tooth Ex Vivo

Recombination and reassociation assays were completed according to the protocol [13]. Essentially, single-cell suspension was prepared from mesenchyme of E10.5 branchial arch and E14.5 tooth germ and epithelium of E13.5 and E14.5 tooth germ. Sufficient numbers of epithelial and mesenchymal cells (2 × 10^5^) were used for this purpose. For the positive control group, we used fresh inductive E14.5 tooth germ mesenchyme cells and E13.5 tooth germ epithelial cells. For the negative control, we used E10.5 branchial arch mesenchyme with E13.5 tooth germ epithelial cells. For the experimental group, in separate groups, we used sEV and secretome purified from E14.5 tooth germ mesenchyme culture for 24 h in 3D well. These were used to treat dissociated E13.5 tooth germ epithelial cells for 24 h or 5 days and were recombined with E10.5 branchial arch mesenchymal cells. In all cases, the desired cell mesenchymal and epithelial cells were mixed and centrifuged to form a pellet. The pellet was then injected into 20 μL of gel-drop collagen gel placed on a cell culture insert (4.0 μm pore size; BD, Franklin Lakes, NJ, USA). The re-association was cultured for 5 days on the cell culture insert containing 1.5 mL/well DMEM 20% FBS, 100 U/mL penicillin/streptomycin, and 0.18 mg/mL.

## Data Availability

RNASeq datasets generated and/or analyzed during the current study are available in the NCBI repository with accession number PRJNA1113680. The mass spectrometry proteomics data have been deposited in the ProteomeXchange Consortium via the PRIDE partner repository with the dataset identifier PXD053767.

## Supplementary Materials

The following supporting information can be downloaded at: https://www.mdpi.com/article/10.3390/bioengineering12020096/s1. Figures S1–S4, Tables S1 and S2.
