# Correction: Birjandi, A.A.; Sharpe, P. The Secretome of the Inductive Tooth Germ Exhibits Signals Required for Tooth Development. *Bioengineering* 2025, *12*, 96

**DOI:** 10.3390/bioengineering13020202

**Published:** 2026-02-11

**Authors:** Anahid A Birjandi, Paul Sharpe

**Affiliations:** Centre for Craniofacial and Regenerative Biology, Faculty of Dentistry, Oral & Craniofacial Sciences, King’s College London, London SE1 9RT, UK; anahid.ahmadi_birjandi@kcl.ac.uk

## Error in Figure

In the original publication [1], an error was identified in Figure 7, affecting panels D–I. The updated Figure 7 includes replacement microscopic images for panels D–I that correspond to the appropriate treatment conditions used in this study. This correction does not affect the figure legend, the manuscript text, or the interpretation of the results. The scientific conclusions of the study remain unchanged.

In addition, the Acknowledgments Section has been updated to thank Dr. Ana Angelova and Xuechen Zhang for their support during the recombination experiments.

The corrected Figure 7 appears below. The authors state that the scientific conclusions are unaffected. This correction was approved by the Academic Editor. The original publication has also been updated.

**Figure 7:**
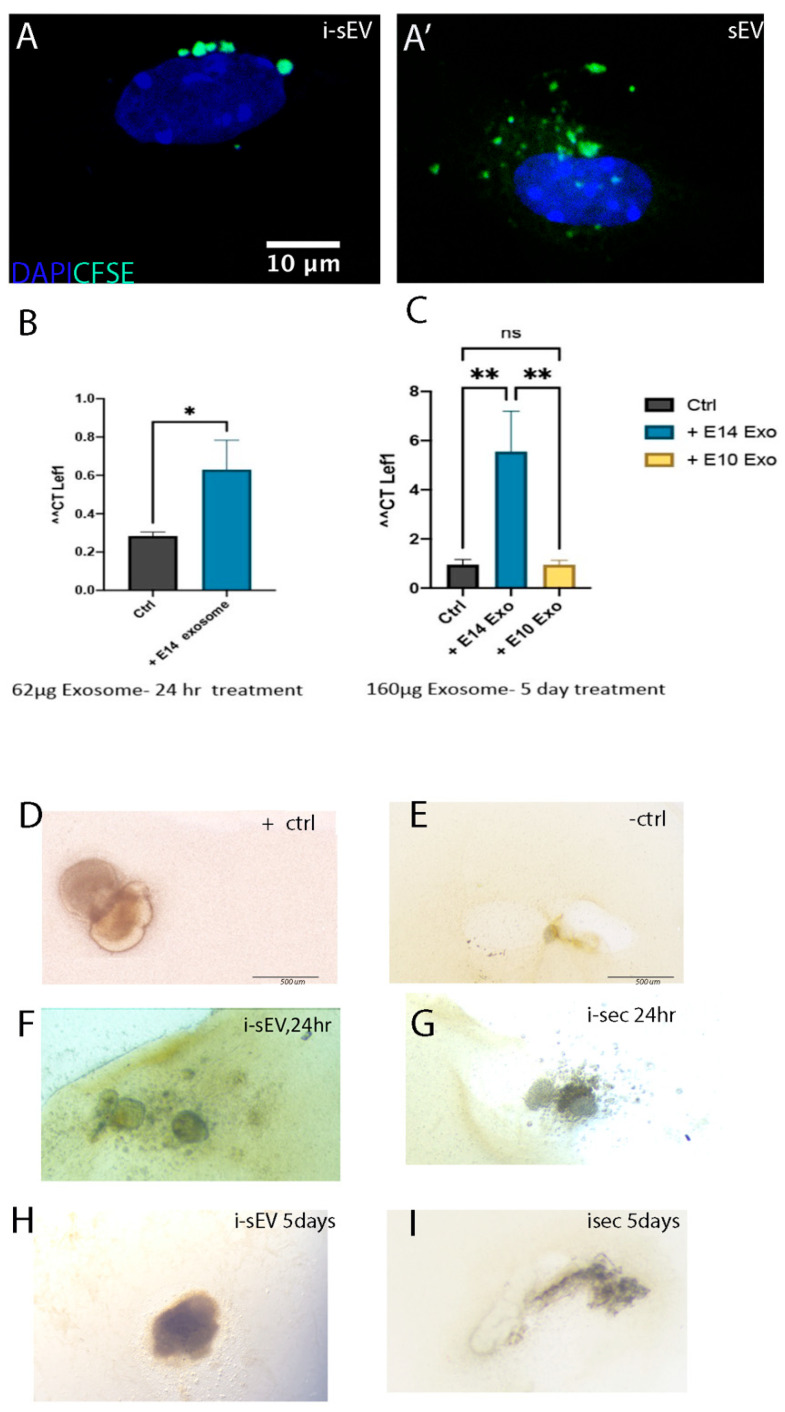
Generation of bioengineered teeth using i-sEV and i-Sec. CFSE-labelled i-sEV and s-EV are taken up by E10.5 branchial arch mesenchyme after 24 h. (**A**,**A′**) Expression of *Lef1* after treatment of tooth germ epithelium with i-sEV for 24 h (**B**) and 5 days (**C**). Recombination of fresh E14.5 mesenchyme cells with E13.5 tooth germ epithelial cells (**D**). Recombination of E10.5 branchial arch mesenchyme with E13.5 tooth germ epithelial cells (**E**). Recombination of i-sEV-treated epithelial cells for 24 h with E10.5 branchial arch mesenchyme (**F**). Recombination of i-sEV-treated epithelial cells for 5 days with E10.5 branchial arch mesenchyme (**G**). Recombination of i-Sec-treated epithelial cells for 24 h with E10.5 branchial arch mesenchyme (**H**). Recombination of i-Sec-treated epithelial cells for 5 days with E10.5 branchial arch mesenchyme (**I**). *p* < 0.002 (**), *p* < 0.033 (*), *p* > 0.12 (ns).
