# Supplementary material for: The Secretome of the Inductive Tooth Germ Exhibits Signals Required for Tooth Development
Source: Bioengineering (Basel). 2025 Jan 21;12(2):96. doi: 10.3390/bioengineering12020096 (PMC11851894; doi:10.3390/bioengineering12020096)
Supplement: Supplementary file 1 [file bioengineering-12-00096-s001.zip › bioengineering-3357324-supplementary.pdf]

Supplementary Materials

The Secretome of the Inductive Tooth Germ Exhibits Signals Required for Tooth Development

Anahid A Birjandi and Paul Sharpe *

Centre for Craniofacial and Regenerative Biology, Faculty of Dentistry, Oral & Craniofacial Sciences, King's College London, London SE1 9RT, UK

* Correspondence: paul.sharpe@kcl.ac.uk

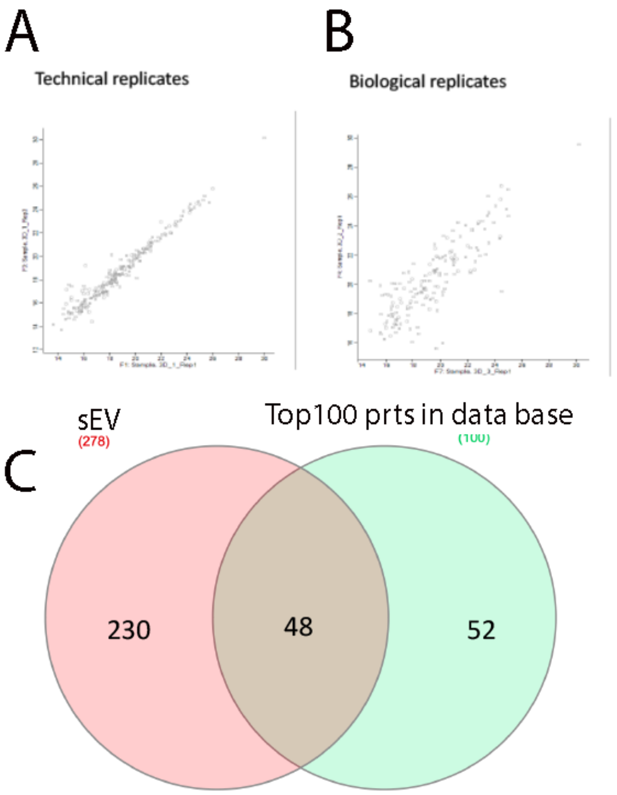

Supplementary Figure S1. PCA demonstrating the biological (**A**) and technical replicates (**B**) in the proteomic analysis of inductive mesenchyme secretome. Venn diagram showing the number of shared and unique proteins identified in sEV and the top 100 proteins identified in sEV databases (**C**).

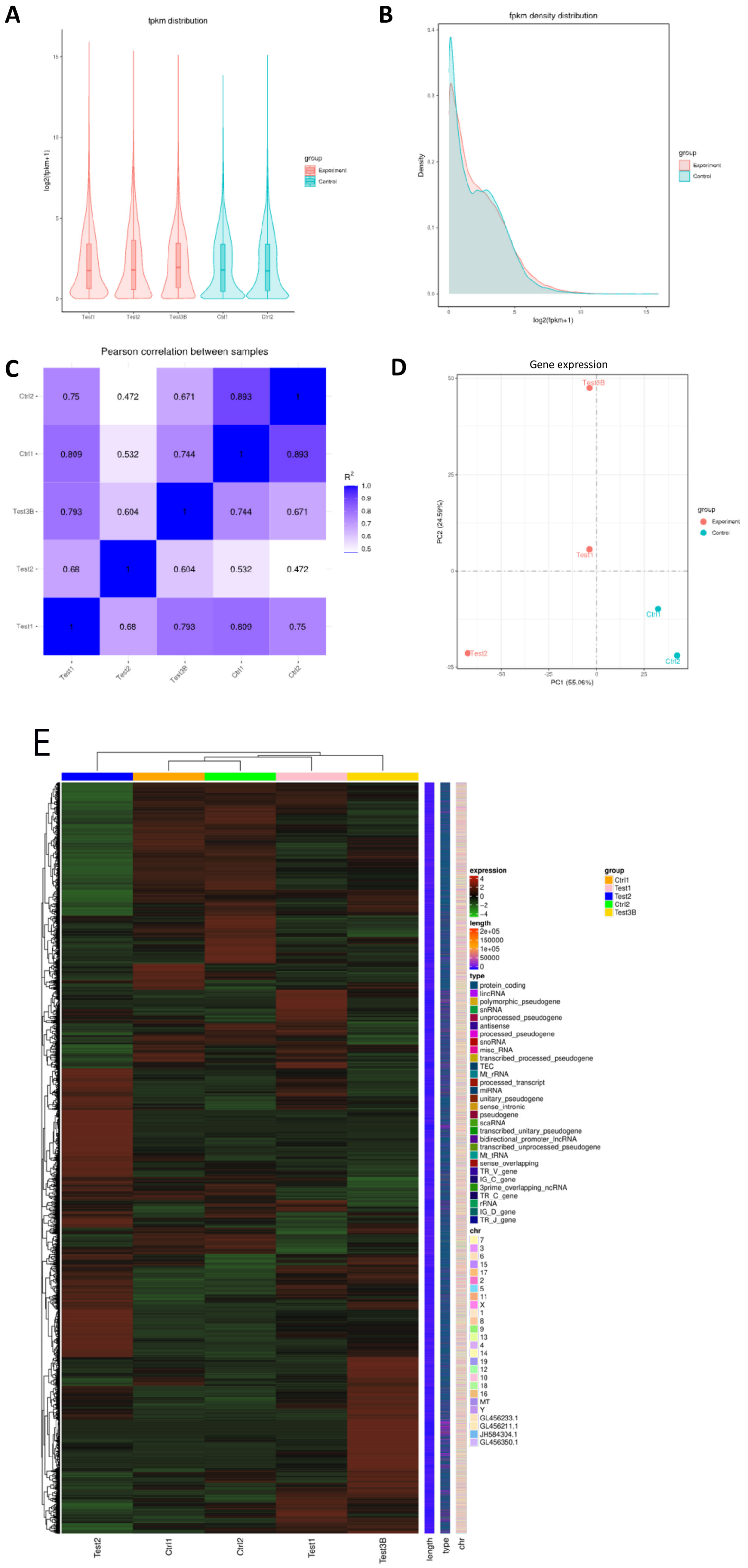

Supplementary Figure S2. Gene expression level analysis. FKPM distribution for different samples: (**A**) co-expression Venn diagram, the number of genes that are uniquely expressed within each group/sample, with the overlapping regions showing the number of genes that are co-expressed in two or more groups/samples. (**B**)

Correlation analysis using fkpm; heat map of sample–sample correlation. (**C**) PCA of treated and untreated epithelial cells (**D**). Heat map of gene expression analysis across all the samplesCtrl1: non-treated E 14 epithelial cells, Ctrl2: E10.5 branchial arch epithelial cells, Test1: pooled inductive Ev-treated E14 epithelial cells, Test2: pooled long-culture inductive Ev-treated E14 epithelial cells, Test3: secretome-treated E14 epithelial cells (**E**).

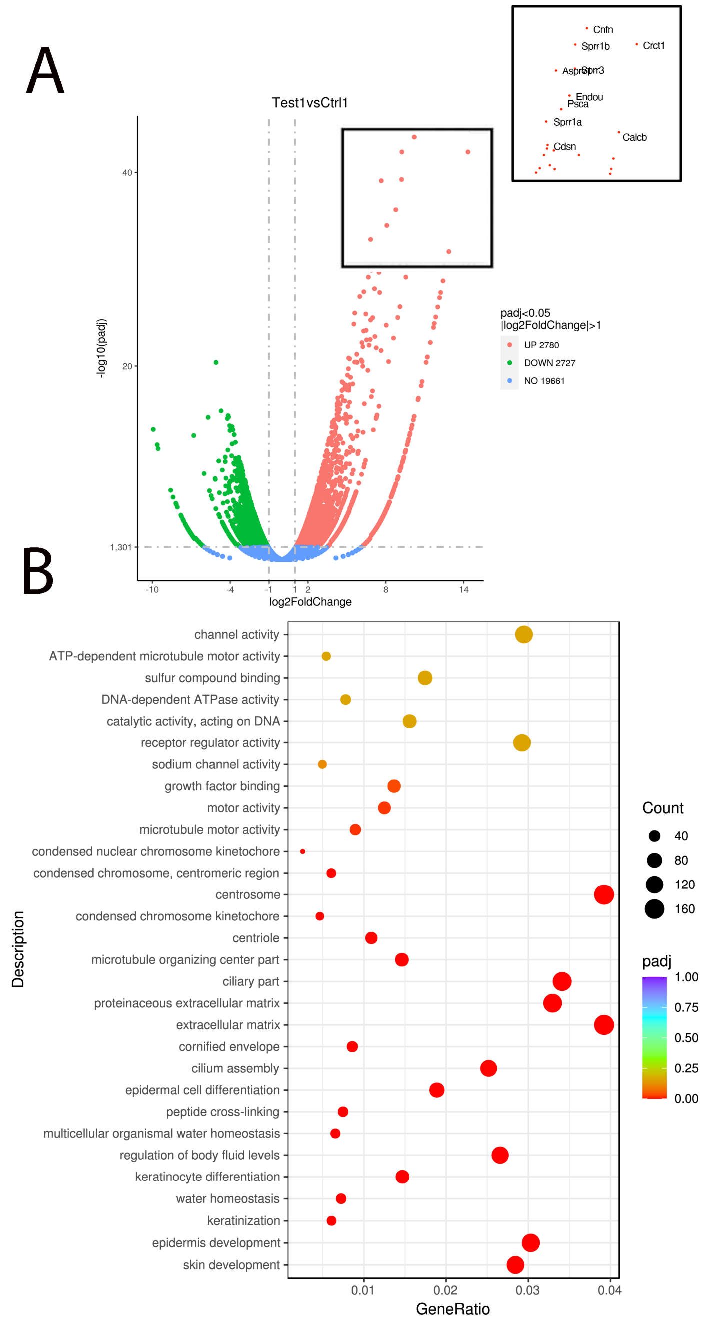

Supplementary Figure S3. Volcano plot demonstrates differentially expressed genes between tooth germ epithelium treated with sEV derived from inductive tooth mesenchyme and untreated epithelium (**A**) and the associated dot plot (**B**).

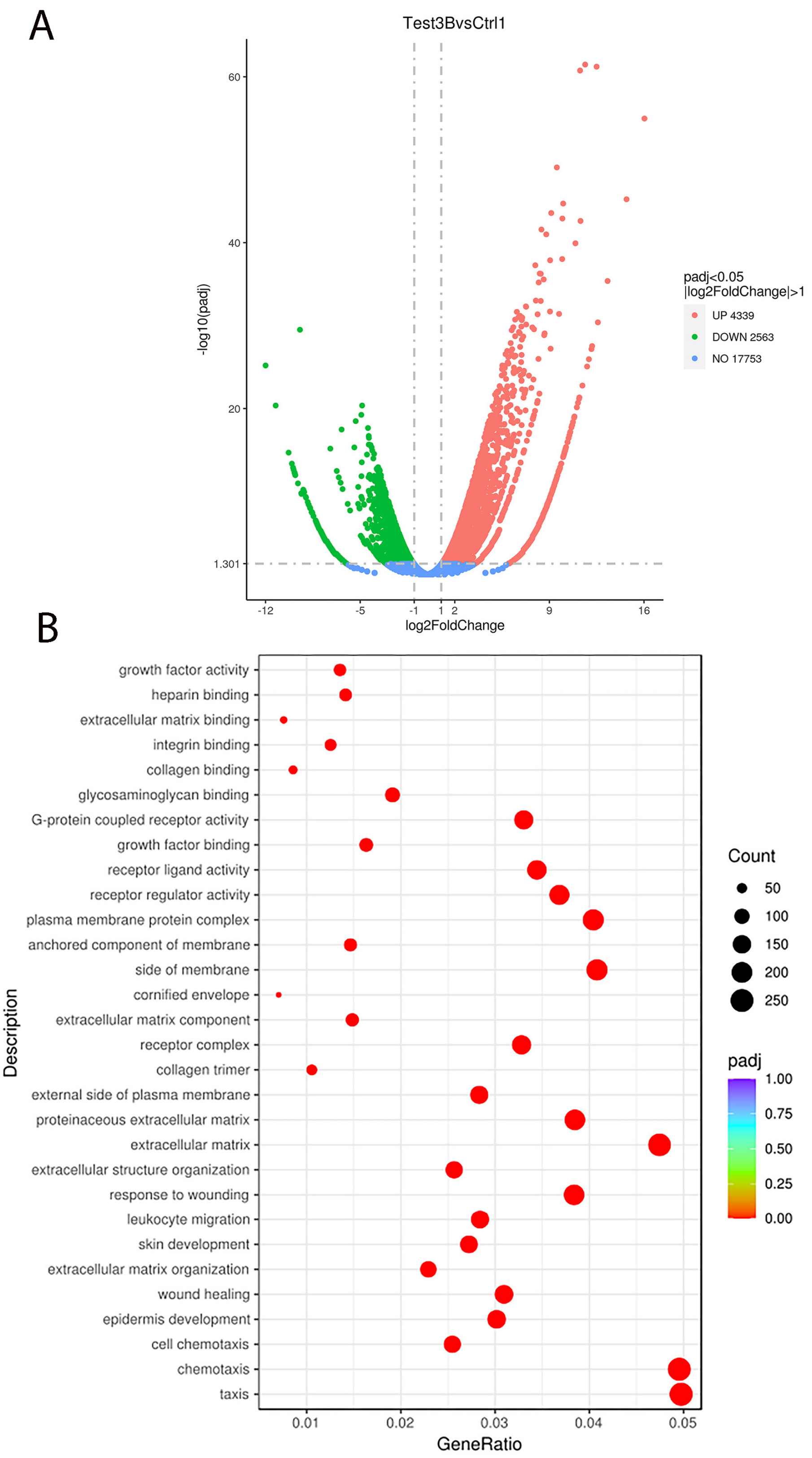

Supplementary Figure S4. Treatment of tooth germ epithelium with secretome from inductive mesenchyme. Volcano plot demonstrates differentially expressed genes between tooth germ epithelium treated with secretome derived from inductive tooth mesenchyme and untreated epithelium(**A**) and the associated dot plot (**B**).

Table S1. Table demonstrating selected normalized counts in different groups for genes that are upregulated only in i-sEV-treated epithelium and present in inducible epithelium.

gene_name	Untreated epithelium	Inductive epithelium	i-sEV treated Epithelium	sEV treated epithelium
Rps26-ps1	80.5278515	393.13253	862.621098	98.0000437
Rab26os	4.58873148	9.77515584	56.8643032	4.02432958
Tgif2-ps2	0.39225998	6.62727343	6.43023202	0.51601956
Rpl30	24.6530042	61.4261804	172.149102	48.9095296
Gtse1	7.31227973	10.9158652	51.0882085	5.90836746
Rpl21	2.48741862	10.1172532	17.5734796	3.94135737
Aen	4.84052438	6.62287257	30.5449184	6.30954662
Cd79a	0.84785501	1.87800764	7.40754974	2.0591199
Fxyd4	0.43700809	1.11284482	3.83773816	0.45990869
Nkx2-9	0.037589	2.34147552	1.93659141	0
Snhg12	4.27256368	8.1368417	23.2537558	5.20423553
Rny1	0	1.99435651	15.8951399	0
Caskin1	0.39260159	0.84142974	2.30752516	0.307856
Hbb-bh1	3.10032565	3395.42937	18.9678545	7.66756237
Cfap73	0.77381906	1.57642947	6.04049832	0.32574785
Hhipl2	0.66991581	1.09019054	3.96229173	0.94002868
Slc5a5	0.14604249	1.67831094	1.33002366	0.47816402
Rps10	3.68011069	5.14683782	17.8381849	4.06768994
Rab39b	0.04191046	0.57467491	0.71974465	0.14702233
Rps2-ps13	341.583563	1115.76332	1512.05672	397.523914
Amhr2	0.30806104	1.72678449	2.27526441	0.89779682
Cox6b2	8.23321136	18.4459977	37.7079198	2.88821921
Ak6	3.79207671	4.90054232	17.1982084	4.6589354
Brsk2	0.05383416	0.21693227	0.49527589	0.06474884
Msx1	1.05856049	49.9819098	5.1914543	1.42079495
Snord118	0.35194437	1.24093294	12.3628866	0
Rgs14	0.25779365	1.15925795	1.52239367	0.28309863
Aqp10-ps	0.17070356	0.66876625	2.53180072	0.11976598
Nme2	1.17665433	1.90749726	6.08114659	0.42700508
Shisa4	1.33616599	3.11772854	5.91301832	2.02656117
Lig4	0.84028242	1.56726824	3.59336495	1.38414657
Mc1r	0.01306006	0.27629348	0.39759696	0.05497778
Ddn	0.03769835	0.23630566	0.51498403	0.07934773
Cuzd1	0.042121	0.6683216	0.73980039	0.04432828
Baat	0.07343507	0.54662425	0.97450883	0
Rap2b	1.54574715	1.85783718	5.85567312	2.59102132
Oxt	0.08847764	1.76780949	2.48639059	0.93114143
Rps27a	137.622386	184.119964	490.851108	83.8273769
Hist1h4d	0.75151567	1.07954623	3.86204409	0.61514247
Hist1h4a	0.88149333	1.34683816	5.47040528	0
Snx32	0.97904526	1.89524303	4.28205435	0.90913264
Spry4	0.83900408	1.74087714	3.25041229	1.59152716
Btbd18	0.28353575	0.57127348	1.39121997	0.42627702
Cubn	0.00636257	0.03365101	0.10057521	0.05356791
Sv2a	0.79939048	1.26475585	2.98805315	0.88442368

Trp53rkb	0.65221316	0.92180509	2.43605793	0.74721066
Nfe2	0.06360441	2.03707365	0.61442057	0.06693748
Snhg8	22.0573813	23.1092845	73.438155	20.9857229
Mthfd2	7.23519716	20.3542183	23.7561002	14.1962352
Gm9755	0.52558065	3.25333082	2.58471854	0.44249819
Zfas1	12.9446799	15.9766409	42.2043369	12.5645917
Yjefn3	1.7817184	2.9824695	7.017343	4.88658792
Slc23a1	0.48422242	2.13848233	1.87275968	1.29715651
Rps2-ps6	6.33499874	9.53558994	21.0169072	4.32768401
CT009567.1	0	3.11250393	2.55364542	0
Six1	0.47696774	1.05746852	1.71354177	1.02674117
Gm5786	2.85505896	3.03901944	9.77677254	2.15429389
Ercc5	4.88574894	9.34191414	14.667224	6.56578567
Met	4.90655098	6.29031314	14.5548478	11.304074
Bmp8b	0.50206498	1.03624297	1.76362312	0.9794264
Phlda2	1.86489704	3.98515484	6.30275617	4.63892835
Snord110	0.6987131	4.1060281	13.0901152	0
Sult4a1	1.05919565	1.66725663	3.3441805	0.43791819
Prcp	1.46072601	2.95616096	4.2304147	1.29167289
Cd101	0.04280405	0.28507919	0.36754528	0.09009423
Mapk12	0.36211885	2.48023521	1.21354484	0.76219004
Rps27l	69.9877618	87.1846634	188.988538	62.3803141
Cdr2	8.34227051	9.59845472	22.2766862	7.1783731
Lmod1	0.04813829	3.25320565	0.33819447	0.22797399
Snora21	1.0404195	4.89126851	8.52768453	0
Slc10a1	0.71301145	1.65062703	2.30222922	1.04597798
Bax	27.4643902	36.7791736	71.906345	41.435912
Nr1h5	0.01675926	1.77276134	0.27473081	0.03527499
Rec8	0.01618824	0.26636721	0.26537031	0.06814623
Snord72	7.62546145	8.96229344	29.5335623	4.93849827
Fv1	1.82475507	2.79209911	5.28110263	1.15947351
Hist1h3e	1.02299621	2.67186518	3.6600651	1.31584987
Snhg20	2.49886802	5.17462375	6.68790142	4.36513774
Fam89a	2.73427636	6.26031191	7.60899633	2.7654483
Neu3	0.32708389	2.85812001	1.09900548	0.47892051
Tusc2	3.99264626	10.5114319	10.6279269	9.07604682
Rpl15	186.034552	228.200191	466.817697	194.994797
Gtf2f2	13.2788828	19.5656298	33.523901	12.6795186
Dok1	1.10550054	2.99405671	3.20797208	1.51752033
Ccnf	4.07819377	11.6405036	10.3762078	2.19435693
Pin4	16.2024161	22.4740389	41.3615467	35.4854997
Snord85	0	1.52991732	7.62095747	0
Ier3ip1	12.4927245	17.0710178	30.900689	13.5697886
Ccm2	4.18617961	5.8187772	10.4103194	4.01000402
F420014N23Rik	0.39097269	1.2472543	1.26438613	0.78373503
Zswim1	2.66520983	3.09229243	6.80158808	4.89055169
Peg10	6.42811857	43.5461925	15.7127523	2.96998782
Palm3	0.74978183	3.47852879	2.04850155	0.52604851

Asb6	5.40728657	7.26900596	13.2152643	10.5722815
Txnl1	37.5010627	46.9133775	89.5173514	67.1649279
Snrpf	61.9704145	78.0879751	145.511606	20.2099868
Snhg6	18.8640342	21.0991085	44.4675553	7.67923553
Rpsa	357.662638	488.626152	826.334168	269.25526
Ldhc	0	0.28274421	0.31689678	0.06329404
Tex21	0	0.09211049	0.20647295	0.16495602
Ttc23l	0	0.15355266	0.22946696	0.04583162
Snrpb2	13.8695895	18.6856615	32.3049106	7.94576196
Pdp1	2.65656703	3.13468416	6.27059212	1.37570104
Nus1	16.6445031	18.1053314	38.1898126	35.15674
Gpx1	108.642371	189.110946	248.553218	125.265985
Frs3	0.71390251	1.80308191	1.88526115	0.4156201
Tbx19	0.01488487	0.45485324	0.20914658	0
Dnaic1	0.01707854	0.40145206	0.23996976	0.03594701
Psph	3.47920062	4.1794415	8.09627706	5.9831806
Foxf2	0.08042741	0.47263633	0.44732408	0.46553131
Cdc34	48.9028052	93.6028137	110.80591	55.0915871
Srp19	10.1438513	11.0299452	22.8662315	12.3146148
Phykpl	4.96728172	7.75535107	11.1678968	2.29931164

Table S2. Table demonstrating selected normalized counts in different groups for genes that are upregulated only in epithelium treated with inductive secretome and present in inducible epithelium

gene_name	Untreated epithelium	Inductive epithelium	i-Sec treated epithelium	i-sEV treated Epithelium	sEV treated epithelium
Thsd4	1.834459095	2.271849555	7.307171064	0.389820747	0.414719449
Arrb1	1.045912544	3.363011546	2.652345825	0.224025461	0.375856177
Hmcn1	0.921505999	3.66025734	8.938647635	0.212650283	0.453043303
Plxna4	0.629996152	1.500681504	1.994586112	0.224851479	0.226912492
Gpc6	3.379487655	7.179062615	9.60843483	1.324801151	1.805530341
Fndc3c1	0.654566378	14.04229101	2.610866765	0.220241447	1.187702969
Vsir	0.217947204	1.124858437	2.924343368	0.033286591	0.139615503
Myo1b	4.820809595	13.74834569	11.84891381	2.185729018	1.389982255
Slc2a3	3.39702831	62.23933835	9.11158376	1.574156363	1.697800493
Ccdc80	0.962866685	8.090778694	8.8245461	0.375811017	1.584046616
Cxcl14	9.648130265	200.3831611	23.21550433	4.719821224	0.383159349
Ass1	5.845571538	10.3171185	16.00618672	3.894770186	0.57997691
Jam3	2.999225966	4.886173443	6.961615889	1.964539943	0.50002295
Nkain1	15.79307189	34.85228903	34.20101149	19.89679148	3.436342854
Cox4i2	3.738292404	11.409339	12.93735724	2.329807937	0.253818756
Nes	7.457150559	12.43737415	26.47252108	7.543750138	2.057451041
Lrp8	1.324059324	2.629045196	4.333385911	0.788407403	0.313881631
Igfbp4	40.35764313	68.601167	116.3546912	21.98359754	11.82119474
Fam171b	0.992600778	2.73182399	2.662248652	1.7143174	0.191512968
Pros1	5.753102452	7.062207632	15.79404049	5.715718102	1.859185137
Ptgis	1.129046328	3.197807318	5.368772727	0.67184366	0.194788839
Fbxo17	2.245283895	4.338544072	5.44367241	2.161153378	0.600748438
Tmem59l	0.62854768	5.775598881	2.043423032	0.468347476	0

Cd276	8.48159003	19.89122548	27.06087249	7.528247626	3.63603348
Tle6	0.430812738	1.193513868	1.222730463	0.342301425	0.064769812
Six2	2.421589891	9.618840729	18.62058657	1.811550862	0.896690519
Plekho1	6.453123716	13.5748465	16.13809054	4.738537321	3.038813509
Spsb4	0.275496154	3.785897099	1.192916636	0.161291004	0.06690762
Crabp1	5.746523171	140.6493816	220.6468448	5.896264476	2.99265606
Slit3	2.983724889	6.295684165	45.56632273	4.649072967	5.587861548
Steap3	0.299291279	2.091020856	5.659833531	0.330974916	0.787437717
Bmp4	2.113544953	14.60635478	29.56135826	1.707596209	3.069535903
Rab3il1	0.355585445	1.770029812	5.513115724	0.318392632	0.638374006
Tbx3	0.824869628	13.42095949	8.417248939	1.125425815	1.16249297
Stard8	0.043201028	8.794172866	1.213050606	0.101169284	0.200045552
Pmp22	1.211023548	7.353871913	12.61805202	1.063502349	1.699313339
Gusb	2.611724415	5.488427011	20.76906807	3.351679668	4.749558364
Acp5	4.07249919	11.66354702	31.61993023	6.193623464	6.749078242
Col11a1	1.230314021	6.918902221	7.30330074	0.977855434	0.863190906
Ank1	0.158058851	2.633783148	1.125824381	0.222087783	0.236574768
Col23a1	0.93600269	1.304537241	5.869504134	0.882601473	0.767120286
Eno3	4.152903142	13.55107331	23.30467543	3.135154096	2.587812852
Gypc	0.631869244	4.000691132	4.400500801	0.859196749	1.544472433
Ddc	0.213012735	3.004276111	1.636373883	0.498838731	0.336262912
Zbtb46	0.083766732	1.312693517	0.665689645	0.163472583	0.176312747
Nr2f1	1.091490555	3.958169169	5.486129658	0.797409824	1.649398304
Fermt3	0.624439381	1.748434696	3.575836717	0.645144834	0.231939521
Trib2	4.581365871	9.120213011	20.64273646	4.786875454	4.3369926
Rgl1	1.561507942	2.069718756	6.951713342	1.01487718	1.04246858
Abcg2	0.582760831	2.561621654	2.724670794	1.050837776	0.785023152
Adra2a	0.549999891	1.704201509	2.698649662	0.922093749	0.552511547
B4galt4	0.621078308	3.128402364	3.055418095	0.259724508	1.05824963
Col4a1	48.0790248	71.32122406	189.1436904	43.96824829	41.77576523
Pcdh7	1.962511398	8.399468278	7.465651199	2.381872618	1.763566421
Uggt2	0.471560355	1.941988284	1.829470799	0.369553306	0.703377079
Pgp	12.81495607	20.11833828	45.95656851	19.88053277	10.41917345
Lama1	0.319007529	8.761901763	1.267567702	0.17239848	0.196269725
Slc25a30	2.608977276	6.841284191	9.168953735	3.833820105	2.975415498
Mum1l1	1.603632116	5.803704675	5.672623828	2.381490393	1.708248811
Mfap4	8.577957451	15.16624074	27.89606748	6.281143979	6.920192753
Bend4	0.49982794	9.433148926	1.65504389	0.804725622	0.495673165
Robo2	0.414052205	1.66049585	1.337671782	0.252236954	0.247317481
Arhgap22	0.821294341	1.483932964	2.825024726	0.703306627	0.490218578
Scd1	6.101169692	13.69491127	18.15284456	3.600729057	4.34263108
Gm14719	3.353822862	11.16839644	12.95614788	3.272528796	7.941540971
Kazald1	0.93272587	5.813412782	3.256929161	2.084996038	0.832875824
Necab2	0.61204067	3.809829893	2.161712787	1.486377583	0.477121136
Rnf128	1.687077465	7.367143726	4.941374778	1.702395176	1.789920491
Cyp4b1-ps2	0	1.173855482	1.88978562	1.169462242	0

Mir207	0	1.413721068	7.965805208	1.408430115	0
Slc1a3	1.803215057	3.61072674	4.585026781	1.994107391	1.792280927
Cep126	1.091058059	2.915493438	2.835660987	0.674923616	0.628278403
Kif5c	2.461442384	12.62738746	5.850661971	3.388455677	2.926485482
Ampd2	7.276032243	13.3832622	16.96118769	11.15002681	7.044733752
Rbm44	0	0.042836746	0.067583465	0.025605856	0
Ifnab	0	0.211122806	0.41636014	0.157749498	0
Slitrk3	0	0.023037121	0.090864073	0.045901807	0
Gm26510	0	0.030903145	0.243779208	0.123149949	0
Gm15191	0.285074943	2.680415146	1.195667362	1.16829278	0.50002295
Miip	1.629080624	3.246626872	3.639950227	2.007033909	1.967889964
